# Neutrophil extracellular traps are associated with poor response to neoadjuvant therapy and poor survival in pediatric osteosarcoma

**DOI:** 10.3389/fonc.2025.1472716

**Published:** 2025-03-19

**Authors:** Szilvia Baron, Yoav Binenbaum, Ronny Maman, Victoria Fidel, Anna Shusterman, Dmitry Vaisman, Osnat Sher, Michal Manisterski, Rachel Shukrun, Claudia Rössig, Ronit Elhasid

**Affiliations:** ^1^ School of Medicine, Tel Aviv University, Tel Aviv, Israel; ^2^ Pediatric Hemato-Oncology Research Laboratory, Tel Aviv Medical Center, Tel Aviv, Israel; ^3^ Dana-Farber/Boston Hematology/Oncology, Boston, MA, United States; ^4^ Department of Pathology, Tel Aviv Medical Center, Tel Aviv, Israel; ^5^ Department of Pediatric Hemato-Oncology, Tel Aviv Medical Center, Tel Aviv, Israel; ^6^ Pediatric Hematology and Oncology, University Children’s Hospital Münster, Münster, Germany

**Keywords:** osteosarcoma, tumor microenvironment (TME), neutrophil, neutrophil extracellular traps (NETs), T cell, chemotherapy resistance, prognosis

## Abstract

**Purpose:**

Osteosarcoma (OS), the most common primary bone malignancy in childhood poses a therapeutic challenge despite extensive research. Neutrophil extracellular traps (NETs) play a role in the tumor microenvironment (TME) in a variety of cancers, but their role in OS has not been characterized.

**Experimental Design:**

This retrospective cohort study aimed to investigate immune cell infiltration and NETs formation in patients with OS and its association with chemotherapy response and overall survival using immunofluorescence of paraffin-embedded tissue samples.

**Results:**

As compared to the non-malignant bone tumor Osteoblastoma, OS samples were characterized by a higher proportion of neutrophils exhibiting NETs. High NETs formation on initial diagnostic biopsies, but not Neutrophil to Lymphocyte ratio, the number of tumor-infiltrating neutrophils, CD3^+^ T-cells or CD8^+^ T-cells, was associated with poor response to neoadjuvant chemotherapy. The NETs burden in diagnostic biopsies was also correlated with survival: patients with high NETs burden had a mean overall survival of 53.7 months, as compared with 71.5 months for patients with low NETs. Furthermore, metastatic sites exhibited elevated NETs formation compared to primary tumors, and sera from patients with OS induced NETs release in healthy neutrophils, while sera from healthy controls did not.

**Conclusions:**

These data highlight the potential role of NETs in OS’s TME biology, and suggest that NETs released by tumor infiltrating neutrophils can serve as an independent prognostic factor for poor response to neoadjuvant therapy and overall survival in patients with OS. Such insights may inform the development of tailored treatment approaches in OS.

## Introduction

Osteosarcoma (OS) is the most common primary bone malignancy in childhood, accounting for 3% of childhood cancers (1). According to the current treatment standard, patients are treated with surgery and intensive (neo)adjuvant chemotherapy. Despite extensive research, long-term event-free survival remains limited to 70% in patients with local disease, and patients with metastatic disease have a disappointingly poor cure rate of 20-30% (2, 3). This poor survival rate, even for patients with localized disease, stems from a few reasons. OS is a heterogeneous tumor, both at the intra- and inter-tumor levels, with no identified driver mutation (4). Consequently, due to a lack of specific targets, efforts to improve treatments using targeted therapies have so far failed. Moreover, while poor histological response to preoperative chemotherapy allows to identify patients with the highest risk of relapse, intensification of chemotherapy has failed to improve outcome (5).

The current therapeutic paradigm involves the administration of neoadjuvant chemotherapy with MAP (high-dose Methotrexate, Doxorubicin, and Cisplatin) before surgical resection, followed by adjuvant MAP chemotherapy post-operatively. This standardized approach is employed across all patient groups, despite potential subgroups exhibiting minimal efficacy with this regimen. Presently, the identification of potential non-responders to MAP therapy relies on histological assessment of the excised tumor post-neoadjuvant treatment. This necessitates that non-responsive patient, identified by a limited necrotic response, endure a prolonged period (2-3 months) of potentially ineffective treatment. Consequently, there is a critical need to establish prognostic factors that can predict responsiveness to neoadjuvant MAP therapy at the time of initial diagnosis.

Neutrophils, the most abundant leukocyte in the circulating blood, are the first line of immune defense within the innate immune system (6). They protect the host from pathogens through mechanisms such as phagocytosis, the release of cytotoxic molecules by degranulation, and the release of neutrophil extracellular traps (NETs) (7). NETs are extruded by activated neutrophils and are composed of DNA fibers, histones, and antimicrobial proteins (8, 9). Besides infections, NETs are also formed in non-infectious conditions, including autoimmune diseases, and thrombosis-associated conditions (10–12) as well as during tumor progression and dissemination (13). Neutrophils are commonly encountered within the tumor microenvironment (TME), playing an important role in various human cancers (14, 15). Clinical observations that are strongly supported by functional studies show that cancer cells and/or other cells within the TME modulate neutrophils to infiltrate the tumor tissue and to acquire tumor-promoting activities, such as angiogenesis, migration, invasion, metastasis or immunosuppression (16, 17). Neutrophils also cross-talk with tumor and other cells through the production of cytokines and other molecules in the TME (18). Cancer cells were recently demonstrated to secrete proteins that induce NETs formation *in vitro* and that inhibition of NETs release suppresses lung metastasis of breast cancer in a mouse model (19). Additionally, recent evidence has implicated NETs release as a mechanism of resistance to chemo-, immuno-, and radiation therapy (20). Furthermore, tumor-infiltrating lymphocytes (TILs) and NETs release were implicated in the development of metastatic niche (21–23). The role of tumor-infiltrating neutrophils (TINs) and NETs formation in the pathogenesis and TME of OS is unknown.

In this retrospective study, we set out to examine whether NETs formation in the TME of treatment-naïve OS patients correlates with response to neoadjuvant chemotherapy and disease progression. We characterize NETs formation along the disease course, in primary tumors and metastatic sites, and explore its utility as an independent prognostic factor for survival.

## Materials and methods

### Study population

The study consisted of two cohorts, a main study cohort and an external validation cohort. The main study cohort had a total of 21 patients who were diagnosed with OS between the years 2012 and 2019 in Tel Aviv Medical Center. There were 10 males and 11 females between the ages of 5.4 and 23.0 years. Diagnostic biopsies were taken before the initiation of neoadjuvant therapy. All patients have been treated as per AOST0331 and received uniform standard treatment with MAP and surgery. Mean follow up time was 62.2 month. The patient’s clinical and pathological characteristics are summarized in **Table 1** and **Supplementary Table 1**.

**Table 1:** Clinical data of OS patients in the main study cohort.

Number of patients	21
Age (years)	
Median (range)	13 (5.4 - 23.0)
Gender	
Male (%)	10 (48%)
Female (%)	11 (52%)
Pathologic Subtype	
Osteoblastic	8/21 (38%)
Osteogenic	2/21 (10%)
Chondroblastic	3/21 (14%)
Osteoblastic & Chondroblastic	4/41 (19%)
Fibroblastic & Osteoblastic	1/21 (5%)
Metastatic disease at diagnosis (%)	4 (19%)
Local control by surgery (%)	21 (100%)
Necrosis	
≥90% (good response) (%)	10 (48%)
<90% (poor response) (%)	11 (52%)
Relapse (%)	4 (19%)
Death from disease (%)	6 (29%)

The external validation cohort consisted of OS patients who were diagnosed in Münster University Hospital, Germany, between 2012 and 2017. There were 2 males and 2 females, of whom 2 presented with metastatic disease. Patients were included into the cooperative osteosarcoma study group (COSS) registry, approved by the institutional Ethical Board, and received uniform standard treatment with MAP and surgery. The patient’s clinical and pathological characteristics are summarized in **Table 2** and **Supplementary Table 2**.

**Table 2:** Clinical data of OS patients of the validation cohort.

Patient No.	Age at diagnosis (y)	Gender	Pathologic subtype	Disease Location	Necrosis (%)	Metastasis Location at diagnosis	Status
**1**	17.08	M	Osteoblastic	Prox Humerus	70	Lung	DOD
**2**	13.50	F	Osteoblastic	Distal Femur	>90	Lung, Lynph nodes	CR
**3**	13.08	M	Osteoblastic	Distal Femur	>90	Lung	CR
**4**	11.00	F	Osteoblastic	Distal Femur	30	–	DOD

CR, complete remission; DOD, dead of disease.

Biopsies of 9 patients, diagnosed with non-malignant bone tumors of Osteoblastomas at the Tel Aviv Medical Center, were used as controls (median age: 17.9 years, range 8.6-21.7 years).

Patients from all cohorts, and/or their legal guardians had given informed consent to the use of leftover material and blood samples for research purposes in accordance with the Declaration of Helsinki.

For NETs induction studies, serum samples from 3 patients between the ages of 11.3 and 17.5 were collected upon their diagnosis of OS. These samples were taken from the biobank of our institution, and the clinical characteristics of the patients are summarized in **Table 3**. As controls, serum samples from 3 healthy pediatric controls (median age: 14.2, between the age of 2.7 and 15.8) were used. For these studies, neutrophils from healthy adult donors, aged 51-64, were isolated from the peripheral blood.

**Table 3:** Clinical data of OS patients used for serum samples.

Patient No.	Age at diagnosis	Gender	Necrosis (%)	Relapse	Death
1	11.26	M	95	Yes	No
2	11.74	F	50	Yes	Yes
3	17.50	M	99	Yes	Yes

### Immunostaining of osteosarcoma tissue samples

All antibodies were obtained from commercial suppliers (**Table 4**). 

**Table 4:** Antibodies and conditions used for the immunofluorescent staining.

Antibodies			Antigen retrieval	
Source	Specificity	Host, dilution	Buffer/pH	Temp. (°C)
EMD Millipore 48001	Neutrophil elastase	rabbit, 1:1000	citrate, 6.0	50
Abcam128012	Histone H3	sheep, 1:500	citrate, 6.0	50
Abcam 150081	Alexa Fluor^®^ 488	goat anti-rabbit, 1:500		
Abcam 150179	Alexa Fluor^®^ 647	donkey anti-sheep, 1:500		
Biolegend 301902	CD15	mouse, 1:250	omniprep, 9.0	95
Abcam 11089	CD3	rat, 1:250	omniprep, 9.0	95
Abcam 93278	CD8	rabbit, 1:250	omniprep, 9.0	95
Abcam 150117	Alexa Fluor^®^ 568	goat anti-mouse, 1:500		
Abcam 150151	Alexa Fluor^®^ 647	donkey anti-rat, 1:500		
Abcam 175693	Alexa Fluor^®^ 488	donkey anti-rabbit, 1:500		

Staining was done on 4-µm thick paraffin-embedded tissue samples. To protect NETs from structural deformation, antigen retrieval for NE and H3 was accomplished with Target Retrieval Solution 10mM Citrate 10x at pH=6.0 (Dako, Denmark) for 30 min at 50°C. For T cell markers, antigen retrieval using Omniprep 10x at pH=9.0 (Zytomed Systems, Germany) for 1h at 95°C was used. Sections were permeabilized for 1 min with 0.5% Triton X100 in TBS at RT, and then treated with 10% (5% BSA and 5% human albumin) blocking buffer in TBS for 1h to prevent non-specific binding. Primary antibodies were diluted (**Table 1**) in TBS buffer containing 1% blocking buffer and incubated for 1h at RT. Secondary antibodies conjugated to fluorescent dyes were diluted in TBS buffer containing 1% blocking buffer and were incubated for 1 hour at RT in the dark. Subsequently, sections were stained with DAPI nuclear dye (Invitrogen, USA, MA) for 10 min.

### Microscopy and image analysis

Imaging was performed on an LSM700 Laser Scanning Confocal Fluorescence microscope (Zeiss, Germany). In each section, 10 regions of interest were collected. Image analysis was done using Image J software. Neutrophils not forming NETs were defined as those exhibiting high intensity of NE signal (green) but low intensity of H3 signal. NETs-forming neutrophils were defined as those exhibiting colocalized high-intensity NE (green) and high density of H3 signals (red). The main difference of not NET-forming and NET-forming neutrophils is the intensity of H3 signal, therefore this was used to count the number of NET-forming neutrophils. The percentage of NETs release was calculated as the ratio of NETs-forming neutrophils and the total number of neutrophils (NETs-forming and non-forming neutrophils). CD8^+^ T cells were defined as the co-localization of CD3 and CD8 signals.

### Isolation of neutrophils

Human peripheral blood samples (2 ml) in EDTA-coated vacutainer tubes (Greiner Bio-One) were obtained from healthy volunteers. Neutrophils were isolated using the EasySep Direct Human Neutrophil isolation kit (StemCell Technologies) by immunomagnetic negative selection according to the manufacturer’s instructions. The number of isolated neutrophils was quantified using Beckman coulter DxH800 hematology analyzer and the final concentration was adjusted to 10^7^/ml in RPMI without phenol red.

### Induction of NETs by serum and monitoring by live cell imaging

To study NETs formation induction by serum of OS patients or controls, 25,000 neutrophils from healthy controls were seeded in 96-well plates (Nunc, Thermo Fischer Scientific) in RPMI containing Cytotox Green dye (Sartorius; final concentration of 50 nM). Neutrophils were then stimulated with serum (final serum concentration 50%) from patients with OS or from healthy controls, and placed in the IncuCyte S3 Live-Cell Analysis System (37°C, 5% CO2; Sartorius). Four pictures per condition were taken every 30 min with a 10X objective for 4 hours. Since the fluorescent dye Cytotox Green (Sartorius) only binds extracellular DNA, NETs formation could be studied over time by measuring the increase in fluorescent particles. The images were processed by the basic analyzer unit of the IncuCyte S3 2021 Software (Sartorius). The proportion of neutrophils producing NETs was calculated by dividing the number of green cells by the number of total cells (as seen in the phase channel) and is expressed as a percentage.

### Statistical analysis

The data are presented as mean ± standard error of the mean. Statistical differences were determined by employing Student’s t-test or one-way ANOVA test with Tukey’s multiple comparison *post-hoc* test. For multifactor response predictions, nominal logistic regression was used, and the probability of ChiSq was calculated. To create Kaplan-Meier curves, parameters (NETs, TINs, TILs, CD8s) were first partitioned (separately for overall survival or progression free survival (PFS) to obtain a minimal sum of squared errors. Kaplan-Meier survival analysis was then calculated. To address multiplicity, parametric survival fit was done for survival analyses, after excluding metastatic disease at presentation as a competing cause. False-Discovery Rate (FDR) approach was used to guards against incorrect declarations of significance, in cases that multiple parameters were evaluated simultaneously. Statistical significance was determined at *P* < 0.05, and statistics was calculated using JMP Pro version 17 (JMP Statistical Discovery LLC).

## Results

### NETs release is elevated in diagnostic biopsies of patients with osteosarcoma compared to patients with osteoblastoma

**Figure 1:**
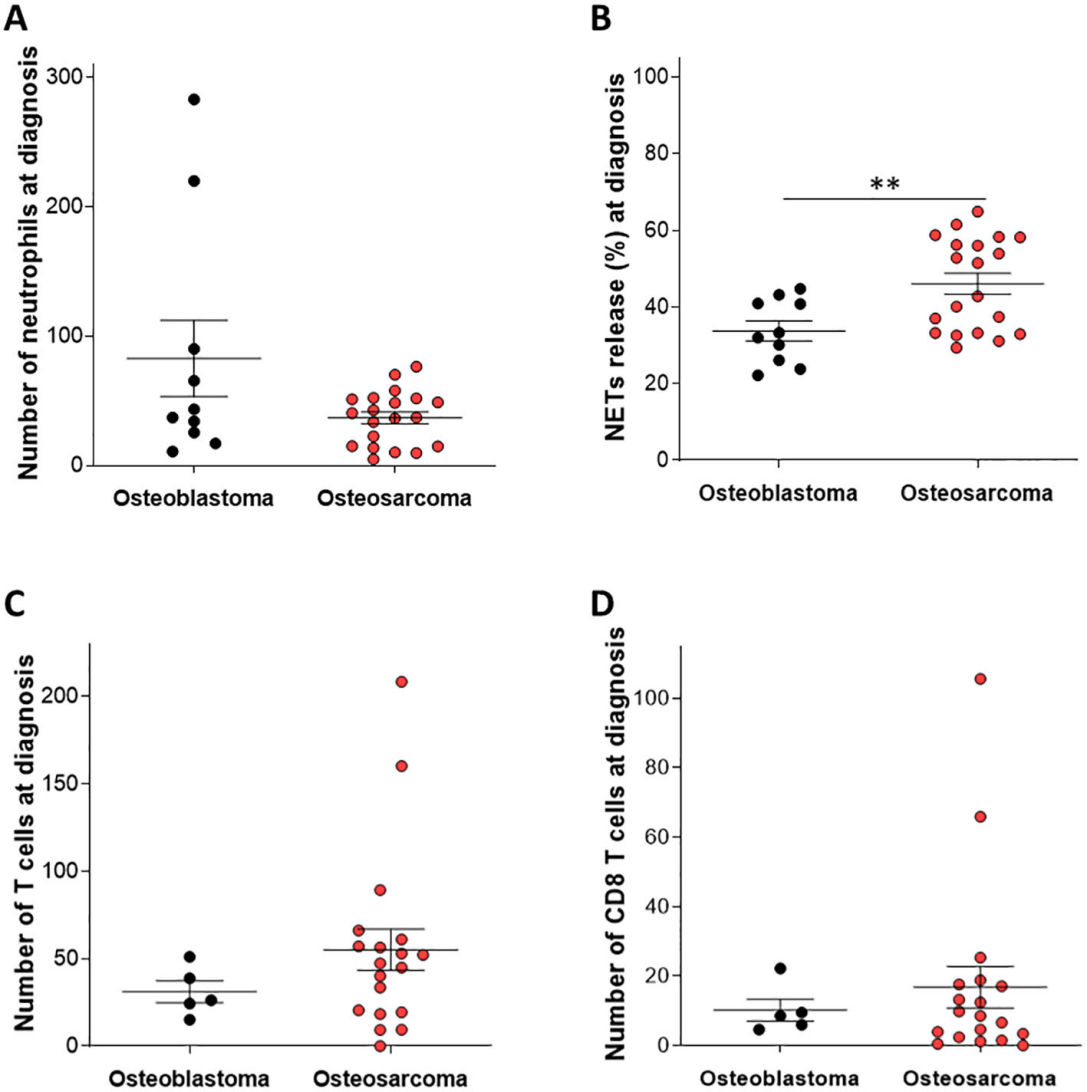
Immune cell infiltration and NETs release in patients with Osteosarcoma at diagnosis compared to patients with Osteoblastoma. Tissue samples from patients with OS at diagnosis were monitored for immune cell infiltration and the extent of NETs-forming neutrophils. The number of infiltrating **(A)** neutrophils **(C)** CD3^+^ T cells and **(D)** CD8^+^ T cells was not significantly changed in tissue samples from patients with OS at diagnosis compared to Osteoblastoma (OB) tissue samples used as controls (*P* = 0.965, *P* = 0.337, and *P* = 0.638 respectively). **(B)** The extent of NETs-forming neutrophils was significantly elevated in patients with OS compared to OB samples (**P = 0.0018).

First, we characterized immune cell infiltration in OS diagnostic biopsies compared to controls having the non-malignant bone condition Osteoblastoma (OB). The numbers of infiltrating immune cells, such as neutrophils, CD3^+^ T cells, and CD8^+^ T cells, did not differ between patients with OS and controls (**Figures 1A, C, D**; *P* = 0.965, *P* = 0.337, and *P* = 0.638 respectively). However, the extent of NETs-forming neutrophils was significantly elevated in diagnostic OS biopsies compared to controls (**Figure 1B**; *P* = 0.0018). Correlation between number of neutrophils and NETs was not observed (R square = 0.01030, P = 0.67).

### NETs release is elevated in diagnostic biopsies of patients with osteosarcoma and poor response to neoadjuvant chemotherapy

**Figure 2:**
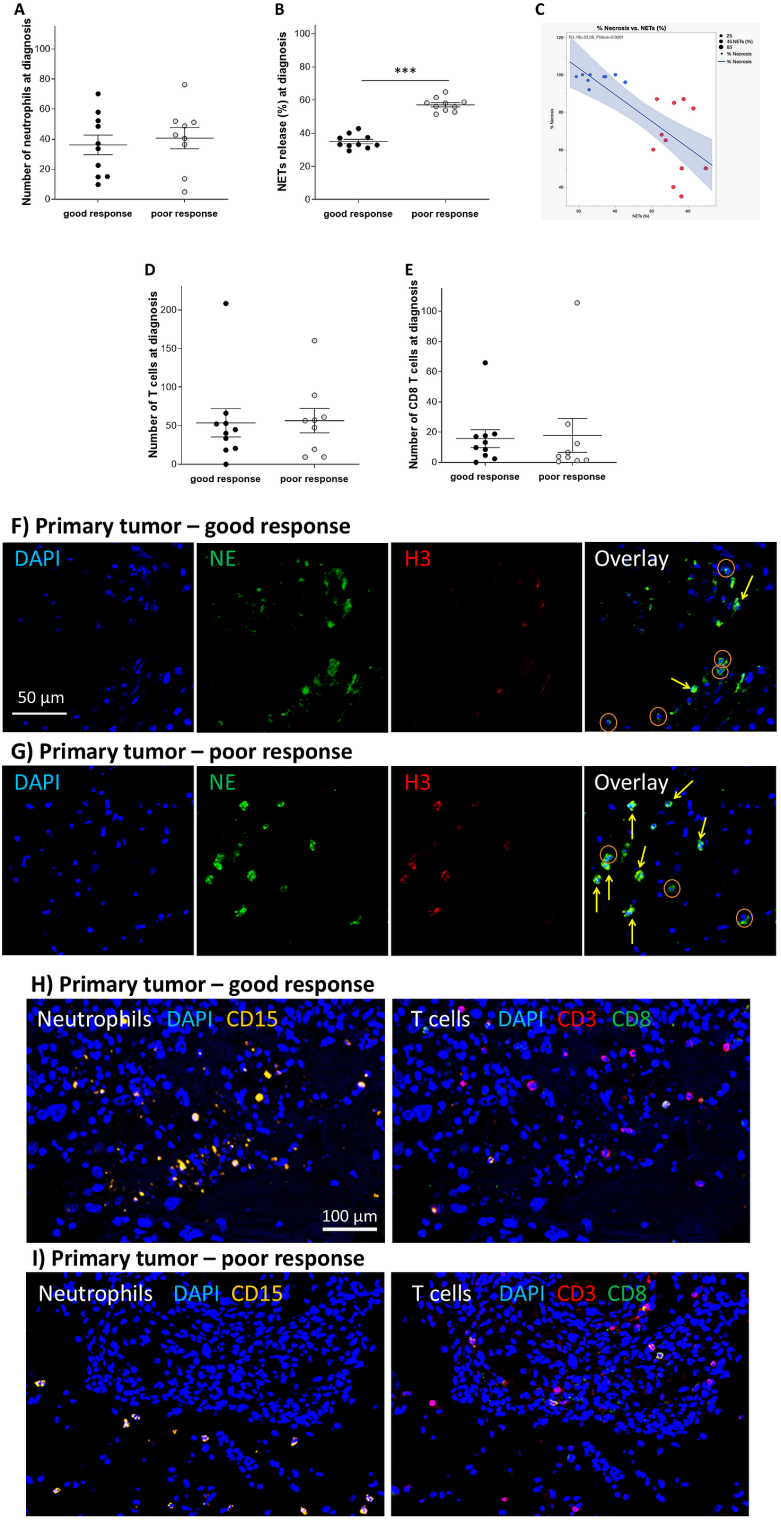
Increased NETs predict poor response to neoadjuvant chemotherapy. Tissue samples from patients with OS at diagnosis with good and poor response were monitored for immune cell infiltration and the extent of NETs-forming neutrophils. **(A)** Although the number of infiltrating neutrophils was not significantly changed in tissue samples from patients with OS at diagnosis with good response compared to patients with OS with poor response (*P* = 0.643), **(B)** however, the extent of NETs-forming neutrophils was significantly elevated comparing the same samples (***P < 0.001). **(C)** NETs forming neutrophils in primary tumor at diagnosis are correlated with the extent of necrosis following neoadjuvant chemotherapy with MAP (F ratio 28.58, P < 0.0001). Circle size represents NETs burden in the sample. Red dots represent cases with >50% NETs forming neutrophils, blue dots represent cases with <50% NETs forming neutrophils. The blue shaded area represents the confidence interval. Furthermore, **(D)** the number of infiltrating CD3^+^ T cells and **(E)** CD8+ T cells was not significantly changed in tissue samples from patients with OS at diagnosis with good response compared to patients with OS with poor response (*P* = 0.906, and *P* = 0.872 respectively). **(F, G)** Representative images of NE (green), and H3 (red) staining for evaluating the number of TINs and the extent of NETs release in primary OS tissue samples from a patient with good and poor response. High H3 signal and co-localization with NE (yellow arrows) represent NETs-forming cells, but cells without NETs had no H3 signal (oranges circles). **(H, I)** Representative images of CD15 (orange), CD3 (red), and CD8 (green) staining for evaluating the number of T and cells CD8 T cells in primary OS tissue samples from a patient with good and poor response.

Next, we characterized the immune cell infiltration in patients with OS who had a good response to neoadjuvant chemotherapy compared with poor response. Good or poor response to chemotherapy was determined by the percentage of tissue necrosis at the time of definitive surgery (Sazer-Kuntschik score) (24), where below 90% was determined as poor response, and 90% and above good response. Overall, no differences were observed in infiltrating immune cells, including neutrophils, CD3^+^ T cells, and CD8^+^ T cells, between samples from OS patients having good or poor response to neoadjuvant chemotherapy (**Figures 2A, D–E, H–I**; *P* = 0.643, *P* = 0.906, and *P* = 0.872 respectively). The extent of NETs-forming neutrophils, however, was significantly elevated in patients having poor response compared to those with good response (**Figures 2B, F, G**; *P* <0.001). Regression analysis confirmed that NETs are correlated with the extent of necrosis following neoadjuvant therapy (**Figure 2C**). Furthermore, multivariate analysis of clinical and pathological parameters showed that only NETs were correlated with response to neoadjuvant therapy, with lL-R ChiSquare of 149.63 (P < 0.0001) and FDR Longworth of 5.8 (**Supplementary Table 3**). The extent of NETs release was not correlated with any of the other immunologic parameters.

### Compared to primary tumor tissue, metastatic tissue exhibits elevated infiltration of immune cells and increase in NETs formation

**Figure 3:**
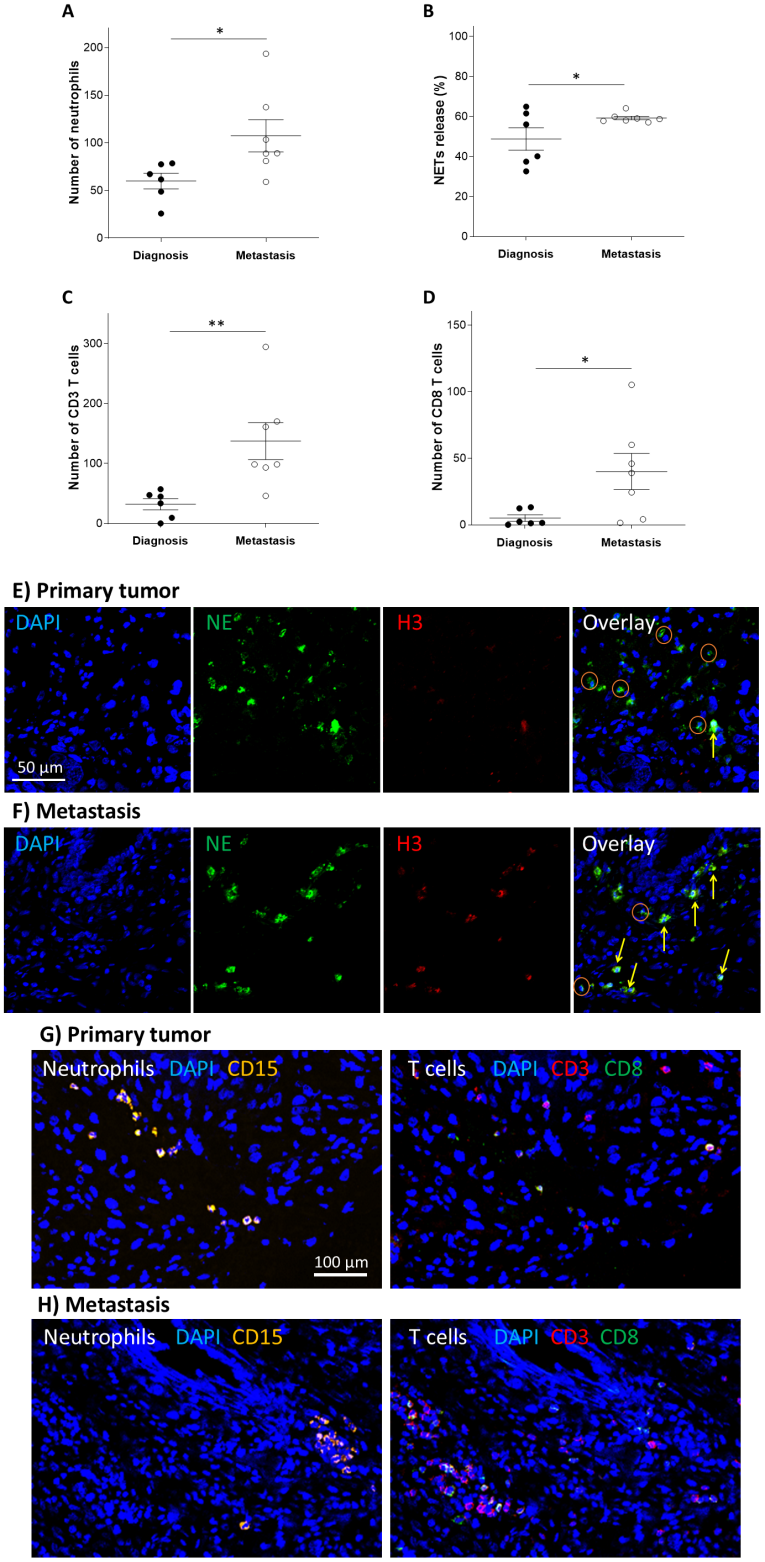
Increased immune cell infiltration in metastatic OS tissue. Immune cell infiltration and the extent of NETs-forming neutrophils in primary disease site compared to metastatic site, in patients with OS. The number of infiltrating **(A)** neutrophils **(B)** NETs-forming neutrophils **(C)** CD3+ T cells and **(D)** CD8+ T cells were all increased in metastasis tissues compared to tissues from diagnostic tissues (*P = 0.034, *P = 0.035, **P = 0.006, and *P = 0.019 respectively). **(E, F)** Representative images of NE (green), and H3 (red) staining for evaluating the number of TINs and the extent of NETs release in primary and metastatic OS tissue samples. High H3 signal and co-localization with NE (yellow arrows) represent NETs-forming cells, but cells without NETs had no H3 signal (oranges circles). **(G, H)** Representative images of CD15 (orange), CD3 (red), and CD8 (green) staining for evaluating the number of neutrophils and CD3 T cells and CD8 T cells in primary and metastatic OS tissue samples.

Further analysis was performed on patients who developed pulmonary metastatic relapse during their disease course. For these patients, we compared biopsies from the time of diagnosis with samples from metastatic tissue. A significantly higher number of neutrophils were found in the metastatic tissue compared to the tissue at diagnosis (**Figures 3A, E, F**; *P* = 0.034), as well as higher numbers of CD3 T cells and CD8 T cells (**Figures 3B, C, G, H**; *P* = 0.006 and *P* = 0.019 respectively). The extent of NETs-forming neutrophils was also significantly increased in the metastatic tissues compared to those acquired at diagnosis, but in contrast to all other immune indexes, NETs-formation in metastasis had a markedly narrow range (57.1-64.1%) (**Figures 3B–D**; *P* = 0.035).

### NETs release might serve as an independent prognostic factor for patients with OS

**Figure 4:**
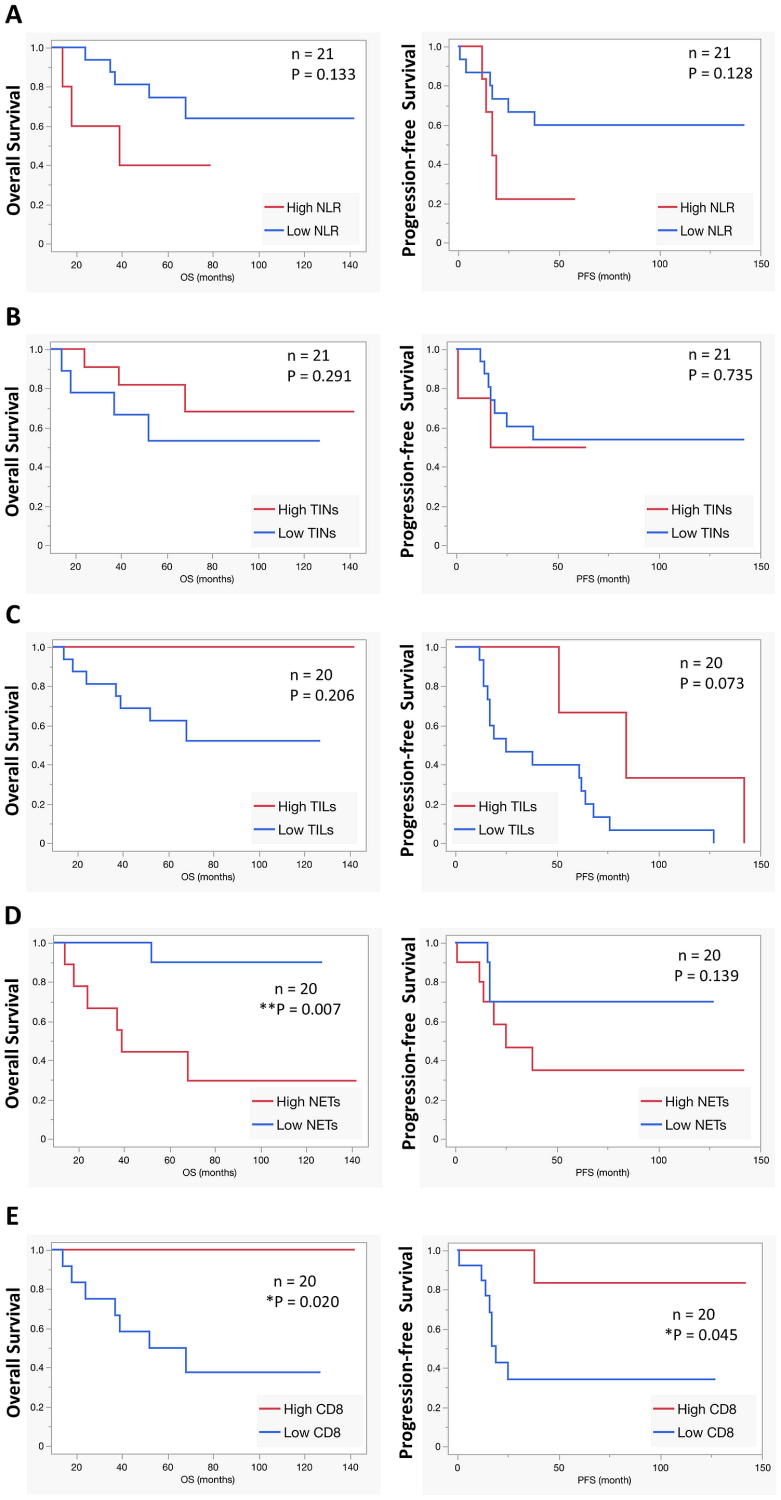
Predictive markers for OS overall survival. The predictive power of prognostic factors such as **(A)** blood neutrophils-to-lymphocytes ratio (NLR), **(B)** number of TINs, **(C)** number of TILs, **(D)** NETs release, and **(E)** the number of infiltrating CD8 T cells were calculated using Log-rank test of Kaplan-Meier analysis for overall survival (OS) as well as progression-free survival (PFS). None of the investigated markers predicted significant differences in overall survival or progression-free survival except increased NETs release, which significantly correlated with shorter overall survival. Conversely, a higher number of infiltrating CD8 T cell was associated with both longer overall and progression-free survival.

We next investigated the prognostic value of the immune-related factors to overall survival and progression-free survival in our patient cohort. Neutrophil-to-lymphocyte ratio (NLR) was recorded at the time of diagnosis in the peripheral blood, and the number of TINs, TILs (CD3^+^), the extent of NETs formation as well as the number of infiltrating CD8^+^ T cells were investigated in the initial biopsy. Log-rank test of Kaplan-Meier analysis and multiple linear correlation was calculated with different cutoff values for each variable. NLR (cutoff 5.5 for overall survival and 4.28 for PFS, n=21) **Figure 4A**, number of TINs (**Figure 4B**; cutoff 40.5 for overall survival and 58.0 for PFS, n=21), number of CD3^+^ TILs (**Figure 4C**; cutoff 89.3 for overall survival and PFS, n=20), did not have predictive value for overall or progression free survival. However, elevated NETs formation at diagnosis (cutoff 52.8 for overall survival and 51.5 for PFS, n=20) and low CD8^+^ TIL count (cutoff 13.0 for overall survival and 17.1 for PFS, n=20) predicted a lower overall survival (**Figures 4D, E**; *P* = 0.007 and 0.02 respectively). This translated to a mean overall survival of 53.7 months in the high NETs group, compared to 71.5 months in the low NETs group. Low CD8^+^ T cells counts were also correlated with poor PFS (**Figure 4E**; *P* = 0.045), but high NETs did not (**Figure 4D**). To guard against multiplicity, survival fit was done after exclusion of cases with metastatic disease at presentation as a competing cause. The analysis, shown in **Supplementary Table 4**, confirm the NETs and CD8s rates were predictive of OS. On FDR analysis TILs, and not CD8s were associated with PFS. However, both Wald test (chi square = 4.29, *P* = 0.0381) and effect likelihood ratio test (chi square=5.78, *P* = 0.0162) confirmed CD8s were significantly associated with PFS.

To validate that our patient cohort was representative of the general OS population, overall survival was calculated for prognostic factors that are known to influence survival, such as response to chemotherapy, development of relapse, and metastatic disease at presentation, using Log-rank test of Kaplan-Meier analysis. As expected, all the markers predicted significantly shorter overall survival (**Supplementary Figures 1A–C**; *P* = 0.033, *P* = 0.047 and *P*= 0.022 respectively).

### Validation of results using external cohort

**Figure 5:**
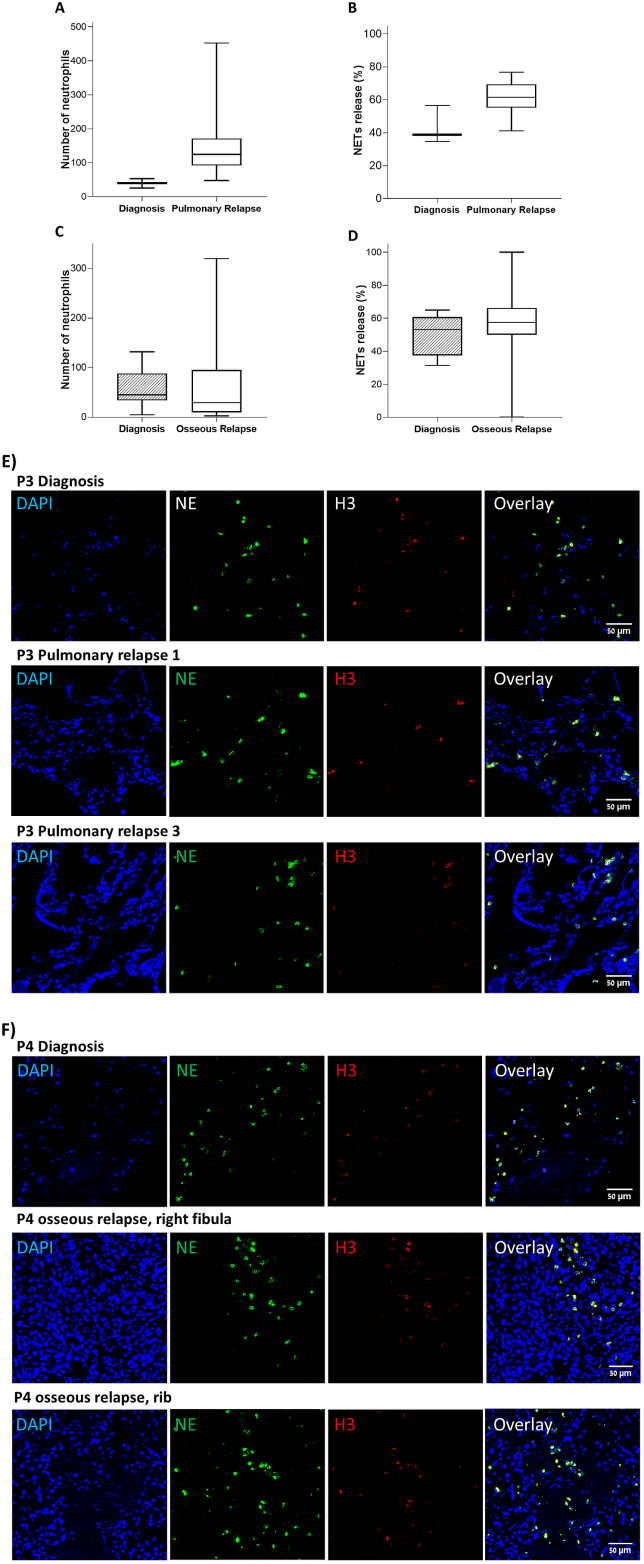
Validation cohort. TINs and NETs release was evaluated in tissue samples in 4 patients at diagnosis compared to samples from relapses. **(A, B)** TINs and NETs-forming neutrophils, grouped for patients with pulmonary relapses. Both TINs and NETs release were significantly elevated in pulmonary metastatic sites compares to the primary disease site (*P* = 0.018 and P = 0.036 respectively). **(C, D)** TINs and NETs-forming neutrophils for patient 4 who had osseous relapse. TINs and NETs were similar in osseous metastatic sites and the primary disease site (*P* = 0.836 and P = 0.336 respectively). **(E)** Representative microscopy images of samples from primary disease site and subsequent pulmonary metastasis in patient 3. **(F)** Representative microscopy images of samples from primary disease site and subsequent osseous metastasis in patient 4.

To validate our previous results regarding NETs formation in primary OS tumors in comparison to metastatic sites of relapse, we gathered a small cohort of 4 OS patients who were diagnosed and treated in Münster University Hospital, Germany. This cohort is small but very informative, as all four patients had long overall survival despite their metastatic disease and underwent multiple surgeries along their disease course to remove subsequential metastasis. This cohort allowed us to investigate NETs in the metastatic niche over the course of the disease. **Supplementary Table 2** demonstrates that in agreement with our previous results, the 2 patients in the Münster cohort who had favorable response to neoadjuvant therapy had low NETs, while the poorly responding patients had >50% NETs. Of the four patients in the cohort, three had pulmonary relapses and one had osseus relapse. **Figures 5A, B** demonstrate that in agreement with our previous data, both TINs and NETs-forming neutrophils were significantly increased in pulmonary metastasis compared to the primary tumor. For the single patient with osseous relapse (who had poor response to neoadjuvant chemotherapy), there was no significant difference in TILs and NETs-forming neutrophils between primary and metastatic sites (**Figures 5C, D**). **Figures 5E, F** show representative microscopy images of the primary disease site and the subsequent pulmonary and osseous relapses. **Supplementary Table 2** shows that with few exceptions (the 3^rd^ relapse of patient 1 and the 1^st^ relapse of patient 2), NETs-forming neutrophils overall increased along the disease course.

### *Ex vivo* NETs formation is induced by serum from patients with OS at the time of their diagnosis

**Figure 6:**
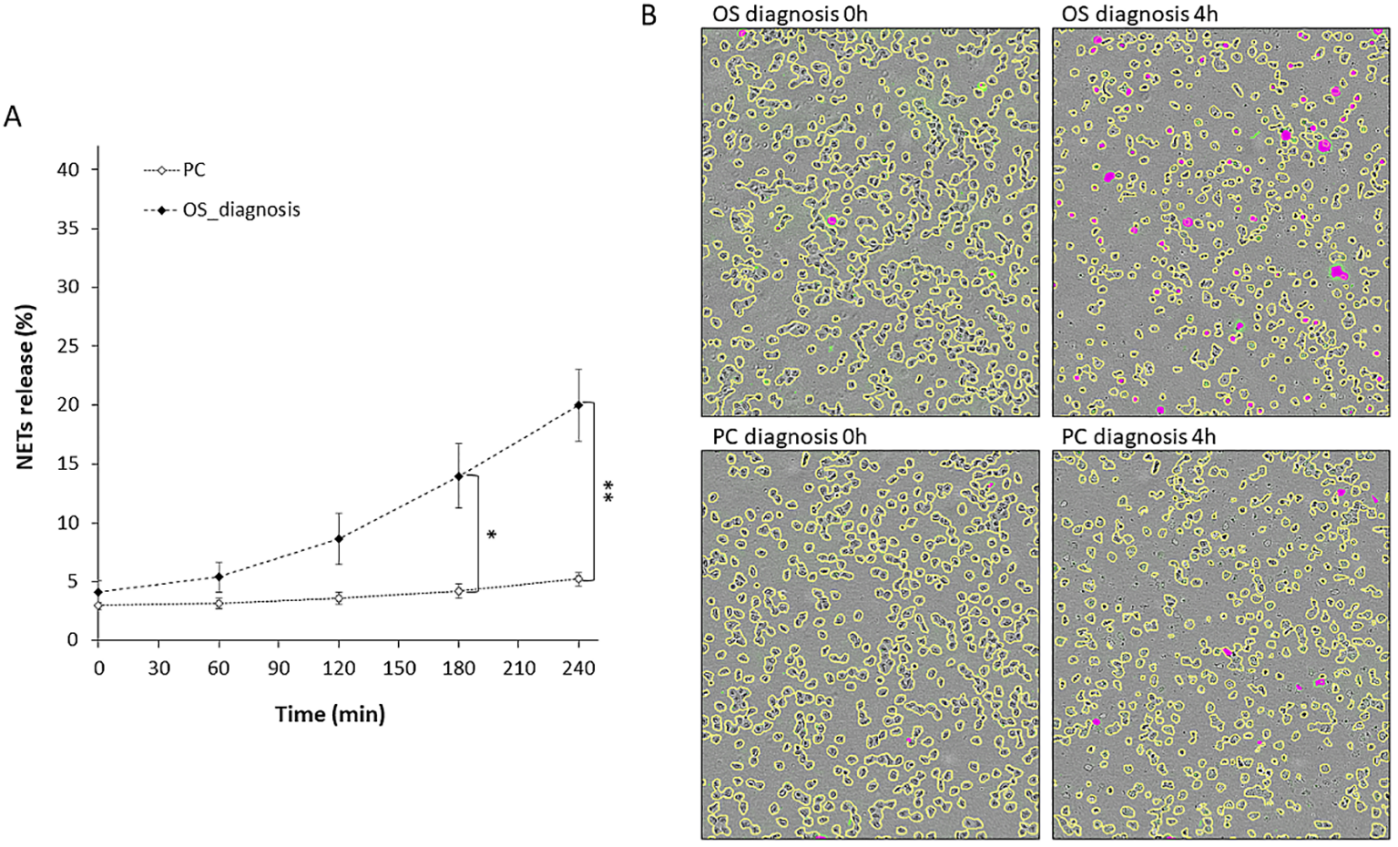
*Ex vivo* NETs formation using serum from patients with OS on freshly isolated neutrophils. Serum samples from patients with OS at diagnosis and from pediatric controls were incubated with freshly isolated neutrophils from healthy volunteers. NETs release was monitored by means of live cell imaging (Incucyte S3, Sartorius). **(A)** NETs release following incubation with serum of patients with OS (*n* = 3, Stable 2) was significantly increased compared to serum of pediatric controls (*n* = 3) (at 3h *P = 0.0247 and at 4h **P = 0.0087). **(B)** Representative images of experimental data at 0 and 180 min. All cells are marked with yellow edges, while NET-forming cells are colored magenta.

To understand whether serum proteins may be involved in inducing NETs release in patients with OS, we applied serum samples from three patients with OS on neutrophils from healthy volunteers and monitored NETs formation, in comparison to serum from three pediatric controls. While serum from healthy pediatric controls caused negligible induction of NETs formation, serum from patients with OS induced a significant increase in NETs formation after three hours of incubation, and an overall fourfold increase compared to baseline levels after four hours of incubation (**Figures 6A, B**, *P* < 0.05).

## Discussion

Osteosarcoma is a highly aggressive malignancy, treated with neoadjuvant chemotherapy using MAP. However, up to 35% of patients show poor response to MAP treatment, highlighting the need for biomarkers for early detection of this group of patients. Recently, the exploration of biomarkers pertaining to the tumor immune microenvironment (TIME) has received increasing attention including in OS (25–27). While the primary focus has been on T cell function (28–30), the role of neutrophils in OS remains relatively underexplored. Neutrophils seem to play a pivotal role in the TIME. Evidence from various solid tumors suggests a potential critical role for neutrophil infiltration in carcinogenesis, with both tumor infiltrating neutrophils and high neutrophil to lymphocytes ratios associated with poor patient outcome (31, 32).

Moreover, NETs, consisting of extracellular DNA webs, have been implicated in promoting metastasis by capturing circulating tumor cells and enhancing metastatic niches through neovascularization, enrichment of growth factors, and the potential reactivation of dormant cancer stem cells, ultimately contributing to the formation of metastatic foci (19, 33, 34).

In our study, NETs exhibited not only greater prevalence in OS tissues compared to OB, but also held prognostic significance. Consistent with prior findings, tumor-infiltrating lymphocytes (TILs) and TINs did not correlate with response to neoadjuvant MAP therapy. Conversely, the presence of NETs demonstrated a strong association with limited necrosis following MAP neoadjuvant treatment, highlighting NETs as a potential biomarker for poor response to MAP neoadjuvant therapy.

Looking into the evolution of the TIME during disease progression by comparing the immune landscape of primary OS biopsies with pulmonary metastases, our analysis revealed a more pronounced infiltration of immune cells within the metastatic lesions, including significant increase in neutrophils, CD3^+^ and CD8^+^ T cells. Additionally, NETs release was significantly elevated in metastases compared to primary tumors. Notably, while other immune cell types exhibited a range of infiltration levels, all metastatic specimens displayed NETs in approximately 60% of their neutrophils. Moreover, in lung metastasis, T-cells and tended to localize in different zones than neutrophils did (can be seen in **Figure 3H**). While we could not determine if the T cells were lung resident or infiltrating, it is plausible that neutrophils inhibited local T cell infiltration in metastatic sites via secreted cytokines or metabolic competition. These findings support the emerging understanding of NETs playing a pivotal role in shaping the metastatic niche within the lung. To strengthen the generalizability of our findings, we employed an external validation cohort that allowed to follow 4 individual patients during the course of disease, with recurring, surgically resected, metastases. Longitudinal analysis of these samples revealed a trend of increasing NETs release within the pulmonary metastatic lesions over time. These findings further emphasize the distinct immune phenotypes observed in pulmonary microenvironments and lend support to the hypothesis that NETs formation correlates with disease aggressiveness. Similar trend was not seen in osseous relapse sites, but these results are limited to a single patient.

We leveraged our findings to develop a prognostic model for survival based on the extent of NETs release, as determined in diagnostic OS samples. Kaplan-Meier survival curves demonstrated a clear stratification of risk based on NETs release, where patients with high NETs had an overall survival of 53.7 months, compared to 71.5 months for patients with low NETs burden at diagnosis. These findings support the role of NETs as an independent prognostic factor for unfavorable treatment outcomes.

To elucidate the potential role of soluble factors in NETs induction, first we employed conditions media from U2-OS cell line, which did not result in NETs release (data not shown). Next, we applied available patient serum samples to stimulate neutrophils from healthy volunteers. This approach was compared to control serum obtained from healthy individuals. Our analyses revealed that incubation with patient serum was sufficient to induce significant NETs release, whereas control serum from healthy individuals did not elicit such a response. These findings suggest that neutrophil activation and subsequent NETs release can be triggered by soluble factors present in the serum, independent of direct cell-cell contact. Few different serum elements could possibly be inducing NETs, including pre-inflammatory chemokines or cytokines (IL-6, IL-8, GM-CSF, TNF), damage-associated molecular patterns (DAMPs, such as HMGB1), complement, and metabolic changes (such as Uric acid). Our findings might open an avenue for future non-invasive screening test for chemotherapy resistance based on induction of NETs formation.

While our findings suggest NETs as an independent prognostic factor in OS, the study’s limitations include the need for larger validation studies and further exploration of the mechanisms by which NETs contribute to chemoresistance. In multiple myeloma, NETs have been shown to internalize chemotherapy drugs, reducing their efficacy. Moreover, degrading NETs through DNase treatment could abrogated the observed effect and restored chemosensitivity in animal models, suggesting a potential mechanism for NETs in chemoresistance (35). This mechanism may also apply to OS.

In conclusion, NETs offer a promise as a prognostic marker and therapeutic target in OS, potentially guiding treatment decisions and improving outcomes. Further research into NETs-targeted therapies may help mitigate their role in OS progression.

## Data Availability

The original contributions presented in the study are included in the article/**Supplementary Material**. Further inquiries can be directed to the corresponding author.

## References

[1] Alfranca A Martinez-Cruzado L Tornin J Abarrategi A Amaral T de Alava E. Bone microenvironment signals in osteosarcoma development, Cell Mol. Life Sci. (2015) 72:3097–113. doi: 10.1007/s00018-015-1918-y PMC11113487 25935149

[2] Andersen ME. Update on survival in osteosarcoma. Orthop Clin North Am. (2016) 47:283–92. doi: 10.1016/j.ocl.2015.08.022 26614941

[3] Xin S Wei G. Prognostic factors in osteosarcoma: A study level meta-analysis and systematic review of current practice. J Bone Oncol. (2020) 21:100281. doi: 10.1016/j.jbo.2020.100281 32140401 PMC7047183

[4] Rickel K Fang F Tao J. Molecular genetics of osteosarcoma. Bone. (2017) 102:69–79. doi: 10.1016/j.bone.2016.10.017 27760307 PMC5393957

[5] Marina NM Smeland S Bielack SS Bernstein M Jovic G Krailo MD. Comparison of MAPIE versus MAP in patients with a poor response to preoperative chemotherapy for newly diagnosed high-grade osteosarcoma (EURAMOS-1): an open-label, international, randomised controlled trial. Lancet Oncol. (2016) 17:1396–408. doi: 10.1016/S1470-2045(16)30214-5 PMC5052459 27569442

[6] Kobayashi SD DeLeo FR. Role of neutrophils in innate immunity: a systems biology-level approach. Wiley Interdiscip Rev Syst Biol Med. (2009) 1:309–33. doi: 10.1002/wsbm.v1:3 PMC3501127 20836000

[7] Mayadas TN Cullere X Lowell CA. The multifaceted functions of neutrophils. Annu Rev Pathol. (2014) 9:181–218. doi: 10.1146/annurev-pathol-020712-164023 24050624 PMC4277181

[8] Brinkmann V Reichard U Goosmann C Fauler B Uhlemann Y Weiss DS. Neutrophil extracellular traps kill bacteria. Science. (2004) 303:1532–5. doi: 10.1126/science.1092385 15001782

[9] Sollberger G Tilley DO Zychlinsky A. Neutrophil extracellular traps: the biology of chromatin externalization. Dev Cell. (2018) 44:542–53. doi: 10.1016/j.devcel.2018.01.019 29533770

[10] Jorch SK Kubes P. An emerging role for neutrophil extracellular traps in noninfectious disease. Nat Med. (2017) 23:279–87. doi: 10.1038/nm.4294 28267716

[11] Lee KH Kronbichler A Park DD Park Y Moon H Kim H. Neutrophil extracellular traps (NETs) in autoimmune diseases: A comprehensive review. Autoimmun Rev. (2017) 16:1160–73. doi: 10.1016/j.autrev.2017.09.012 28899799

[12] Kessenbrock K Krumbholz M Schönermarck U Back W Gross WL Werb Z. Netting neutrophils in autoimmune small-vessel vasculitis. Nat Med. (2009) 15:623–5. doi: 10.1038/nm.1959 PMC2760083 19448636

[13] Albrengues J Shields MA Ng D Park CG Ambrico A Poindexter ME. Neutrophil extracellular traps produced during inflammation awaken dormant cancer cells in mice. Science. (2018) 361:eaao4227. doi: 10.1126/science.aao4227 30262472 PMC6777850

[14] Jensen HK Donskov F Marcussen N Nordsmark M Lundbeck F von der Maase H. Presence of intra-tumoral neutrophils is an independent prognostic factor in localized renal cell carcinoma. J Clin Oncol. (2009) 27:4709–017. doi: 10.1200/JCO.2008.18.9498 19720929

[15] Wislez M Rabbe N Marchal J Milleron B Crestani B Mayaud C. Hepatocyte growth factor production by neutrophils infiltrating bronchioloalveolar subtype pulmonary adenocarcinoma: role in tumor progression and death. Cancer Res. (2003) 63:1405–12. 12649206

[16] Tazzyman S Niaz H Murdoch C. Neutrophil-mediated tumour angiogenesis: subversion of immune responses to promote tumour growth. Semin Cancer Biol. (2013) 23:149–58. doi: 10.1016/j.semcancer.2013.02.003 23410638

[17] Dumitru CA Lang S Brandau S. Modulation of neutrophil granulocytes in the tumor microenvironment: mechanisms and consequences for tumor progression. Semin Cancer Biol. (2013) 23:141–8. doi: 10.1016/j.semcancer.2013.02.005 23485549

[18] Tecchio C Scapini P Pizzolo G Cassatella MA. On the cytokines produced by human neutrophils in tumors. Semin Cancer Biol. (2013) 23:159–70. doi: 10.1016/j.semcancer.2013.02.004 23410636

[19] Xiao Y Cong M Li J He D Wu Q Tian P. Cathepsin C promotes breast cancer lung metastasis by modulating neutrophil infiltration and neutrophil extracellular trap formation. Cancer Cell. (2021) 39:423–37. doi: 10.1016/j.ccell.2020.12.012 33450198

[20] Shahzad MH Feng L Su X Brassard A Dhoparee-Doomah I Ferri LE. Neutrophil extracellular traps in cancer therapy resistance. Cancers (Basel). (2022) 14:1359. doi: 10.3390/cancers14051359 35267667 PMC8909607

[21] Wang Z Yang C Li L Zhang Z Pan J Su K. CD62L^dim^ neutrophils specifically migrate to the lung and participate in the formation of the pre-metastatic niche of breast cancer. Front Oncol. (2020) 10:540484. doi: 10.3389/fonc.2020.540484 33178575 PMC7593663

[22] Kalafati L Mitroulis I Verginis P Chavakis T Kourtzelis I. Neutrophils as orchestrators in tumor development and metastasis formation. Front Oncol. (2020) 10:581457. doi: 10.3389/fonc.2020.581457 33363012 PMC7758500

[23] Rayes RF Mouhanna JG Nicolau I Bourdeau F Giannias B Rousseau S. Primary tumors induce neutrophil extracellular traps with targetable metastasis promoting effects. JCI Insight. (2019) 5:e128008. doi: 10.1172/jci.insight.128008 31343990 PMC6777835

[24] Salzer-Kuntschik M Delling G Beron G Sigmund R. Morphological grades of regression in osteosarcoma after polychemotherapy - study COSS 80. J Cancer Res Clin Oncol. (1983) 106 Suppl:21–4. doi: 10.1007/BF00625047 PMC12253038 6577010

[25] Wu C Gong S Duan Y Deng C Kallendrusch S Berninghausen L. A tumor microenvironment-based prognostic index for osteosarcoma. J BioMed Sci. (2023) 30:23. doi: 10.1186/s12929-023-00917-3 37055822 PMC10099847

[26] Supra R Agrawal DK. Immunotherapeutic strategies in the management of osteosarcoma. J Orthop Sports Med. (2023) 5:32–40. doi: 10.26502/josm.511500076 36937115 PMC10018813

[27] Zhang Z Tan X Jiang Z Wang H Yuan H. Immune checkpoint inhibitors in osteosarcoma: A hopeful and challenging future. Front Pharmacol. (2022) 13:1031527. doi: 10.3389/fphar.2022.1031527 36324681 PMC9618820

[28] Sun CY Zhang Z Tao L Xu FF Li HY Zhang HY. T cell exhaustion drives osteosarcoma pathogenesis. Ann Transl Med. (2021) 9:1447. doi: 10.21037/atm-21-3928 34733999 PMC8506720

[29] Cai X Zhan H Ye Y Yang J Zhang M Li J. Current progress and future perspectives of immune checkpoint in cancer and infectious diseases. Front Genet. (2021) 12:785153. doi: 10.3389/fgene.2021.785153 34917131 PMC8670224

[30] Fritzsching B Fellenberg J Moskovszky L Sápi Z Krenacs T MaChado I. CD8^+^/FOXP3^+^-ratio in osteosarcoma microenvironment separates survivors from non-survivors: a multicenter validated retrospective study. Oncoimmunology. (2015) 4:e990800. doi: 10.4161/2162402X.2014.990800 25949908 PMC4404826

[31] Liu B Huang Y Sun Y Zhang J Yao Y Shen Z. Prognostic value of inflammation-based scores in patients with osteosarcoma. Sci Rep. (2016) 6:39862. doi: 10.1038/srep39862 28008988 PMC5180218

[32] Shen M Hu P Donskov F Wang G Liu Q Du J. Tumor-associated neutrophils as a new prognostic factor in cancer: a systematic review and meta-analysis. PloS One. (2014) 9:e98259. doi: 10.1371/journal.pone.0098259 24906014 PMC4048155

[33] Wang Y Liu F Chen L Fang C Li S Yuan S. Neutrophil extracellular traps (NETs) promote non-small cell lung cancer metastasis by suppressing lncRNA MIR503HG to activate the NF-κB/NLRP3 inflammasome pathway. Front Immunol. (2022) 13:867516. doi: 10.3389/fimmu.2022.867516 35707534 PMC9190762

[34] Park J Wysocki RW Amoozgar Z Maiorino L Fein MR Jorns J. Cancer cells induce metastasis-supporting neutrophil extracellular DNA traps. Sci Transl Med. (2016) 8:361ra138. doi: 10.1126/scitranslmed.aag1711 PMC5550900 27798263

[35] Mousset A Lecorgne E Bourget I Lopez P Jenovai K Cherfils-Vicini J. Neutrophil extracellular traps formed during chemotherapy confer treatment resistance via TGF-β activation. Cancer Cell. (2023) 41:757–775.e10. doi: 10.1016/j.ccell.2023.03.008 37037615 PMC10228050

