# Supplementary material for: Neutrophil extracellular traps are associated with poor response to neoadjuvant therapy and poor survival in pediatric osteosarcoma
Source: Front Oncol. 2025 Mar 19;15:1472716. doi: 10.3389/fonc.2025.1472716 (PMC11961948; doi:10.3389/fonc.2025.1472716)
Supplement: Supplementary file 1 [file DataSheet1.docx]

**Supporting Information**

**Supplementary Figure 1.** **Predictive markers for OS overall survival.** The predictive power of prognostic factors such as response to chemotherapy, development of relapse and metastasis, neutrophils-to-lymphocytes ration (NLR), number of TINs and NETs release were tested in our OS patient cohort using Log-rank test of Kaplan-Meier analysis. All well-known clinical biomarkers **A)** poor response to chemotherapy **B)** development of relapse and C) metastasis predicted poor outcome with significantly shorter overall survival (*P* = 0.033, *P* = 0.047, *P* = 0.022 respectively).


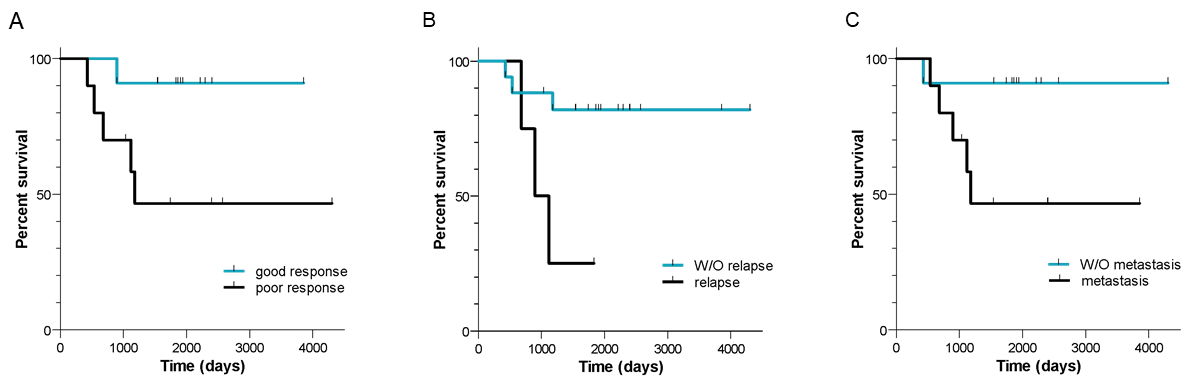


**Supplementary Table 1.** **Clinical information of OS patients used in our study for tissue staining.** Patients identified as good responder if they had no local or metastatic disease progression and had cell death > 90% at biopsy from operation. Abbreviations: M – male, F – female, OB - osteoblastic; OG - osteogenic; ChB - chondroblastic, OB&ChB - osteoblastic and chondroblastic; FB&OB - fibroblastic and osteoblastic, PFS – progression-free survival, NLR – neutrophil to lymphocyte ratio, TIN – tumor-infiltrating neutrophils, TILs – tumor-infiltrating lymphocytes

|  |  |  |  |  |  |  |  |  |  |  |  |  | **Diagnosis** | | | | **Metastasis** | | | |
| --- | --- | --- | --- | --- | --- | --- | --- | --- | --- | --- | --- | --- | --- | --- | --- | --- | --- | --- | --- | --- |
| **Patient no.** | **Gender** | **Age at diagnosis (y)** | **Type of disease** | **Location** | **Spread at diagnosis** | **Relapse** | **Site of relapse** | **Death** | **Overall Survival**  **(month)** | **PFS (month)** | **NLR at diagnosis** | **% Necrosis** | **NETs (%)** | **TINs** | **TILs** | **CD8^+^ T cells** | **NETs (%)** | **TINs** | **TILs** | **CD8^+^ T cells** |
| 1 | M | 13.8 | OB | Femor | Local | Yes | Lung | No | 79 | 17 | 7.06 | 97 | 32.6 | 58.0 | 44.9 | 13 | 59 | 45.2 | 170.1 | 39.0 |
| 2 | F | 11.6 | OB | Femor | Metastatic | Yes | Lung and Skeleton | Yes | 52 | 16 | 1.78 | 99 | 37.4 | 22.6 | 33.5 | 2.4 | 57.1 | 51.5 | 98.4 | 45.9 |
| 3 | F | 7.6 | OG | Femor | Metastatic | No |  | No | 127 | 127 | 2.33 | 100 | 40.1 | 9.8 | 0.0 | 0.0 | 58.7 | 88.0 | 161.0 | 60.0 |
| 4 | M | 13.5 | OB&ChB | Humerus | Metastatic | Yes | Lung and Humerus | Yes | 35 | 4 | 2.09 | 96 | - | - | - | - | 59.9 | 201.7 | 98.5 | 24.4 |
| 5 | F | 14.4 | OB | Tibia | Metastatic | Yes | Lung | Yes | 18 | 12 | 6.5 | 40 | 56.0 | 36.5 | 47.3 | 12.4 | 64.1 | 125.4 | 294.4 | 105.1 |
| 6 | M | 5.4 | N/A | Femor | Local | No |  | No | 142 | 142 | 2.17 | 87 | 58.8 | 52.0 | 160.2 | 105.6 |  |  |  |  |
| 7 | M | 14.6 | OG | Femor | Local | Yes | Local and Lung | Yes | 68 | 14 | 4.28 | 82 | 61.5 | 42.8 | 9.4 | 1.2 | 58 | 49.6 | 45.9 | 4.1 |
| 8 | F | 11.8 | N/A | Femor | Local | Yes | Lung and Skeleton | Yes | 39 | 19 | 5.25 | 50 | 64.9 | 51.2 | 57.0 | 1.5 | 57.8 | 50.0 | 93.2 | 1.5 |
| 9 | F | 23 | OB | Femor | Local | No |  | No | 58 | 58 | 33.8 | 65 | 53.9 | 73.0 | - | - |  |  |  |  |
| 10 | F | 6.2 | OB | N/A | Local | Yes | Local and Lung | Yes | 37 | 25 | 1.81 | 68 | 52.8 | 4.9 | 9.3 | 0.5 |  |  |  |  |
| 11 | M | 10 | ChB | Femor | Local | Yes | Local and Lung | No | 60 | 17 | 1.81 | 99 | 37.0 | 37.1 | 66.1 | 9.8 |  |  |  |  |
| 12 | F | 21 | OB | Femor | Local | No |  | No | 51 | 51 | 1.54 | 100 | 33.2 | 33.7 | 208.3 | 65.9 |  |  |  |  |
| 13 | M | 6.8 | OB&ChB | Femor | Local | Yes | Lung | Yes | 24 | 1 | 2 | 85 | 56.2 | 76.3 | 61.0 | 6.6 |  |  |  |  |
| 14 | F | 13 | FB&OB | Femor | Local | No |  | Yes | 14 | 14 | 14.39 | 35 | 58.2 | 13.7 | 19.3 | 3.4 |  |  |  |  |
| 15 | M | 19.6 | N/A | Tibia | Local | No |  | No | 84 | 84 | 2.32 | 50 | 58.3 | 40.5 | 89.3 | 3.9 |  |  |  |  |
| 16 | F | 17 | ChB | Pelvis | Local | No |  | No | 76 | 76 | 3.56 | 60 | 50.5 | 48.5 | 52.2 | 18.8 |  |  |  |  |
| 17 | M | 10.5 | ChB | Femor | Local | Yes | Lung | No | 87 | 38 | 0.63 | 87 | 51.5 | 48.8 | 56.4 | 25.3 |  |  |  |  |
| 18 | F | 17.3 | OB&ChB | Humerus | Local | No |  | No | 68 | 68 | 0.92 | 96 | 42.8 | 14.9 | 20.5 | 8.5 |  |  |  |  |
| 19 | M | 5.6 | OB&ChB | Femor | Local | No |  | No | 61 | 61 | 0.92 | 92 | 32.9 | 15.1 | 18.3 | 4.6 |  |  |  |  |
| 20 | F | 17.2 | OB | Tibia | Local | No |  | No | 64 | 64 | 2.13 | 100 | 31.1 | 70.2 | 52.9 | 17.1 |  |  |  |  |
| 21 | M | 11.7 | OB | Humerus | Local | No |  | No | 62 | 62 | 4.16 | 99 | 29.4 | 52.2 | 40 | 17.6 |  |  |  |  |

**Supplementary Table 2.** **Clinical information of OS patients from the German cohort.**

| **Samples** | **Slides** | **Time of sample** | **% of necrosis** | **Neutrophils/1mm^2^** | **NETs release (%)** |
| --- | --- | --- | --- | --- | --- |
| **P1** | Osteosarcoma Humerus | 2016.02.25 | 70 | 40.0 | 56.6 |
|  | Pulmonary relapse 1 | 2016.10.16 |  | 154.1 | 56.9 |
|  | Pulmonary relapse 2 | 2017.06.13 |  | 72.2 | 70.1 |
|  | Pulmonary relapse 3 | 2017.09.14 |  | 135.4 | 53.5 |
|  | Pulmonary relapse 4 | 2018.01.31 |  | 124.6 | 68.9 |
|  | Pulmonary relapse 5 | 2018.03.07 |  | 452.4 | 76.8 |
| **P2** | Osteosarcoma left distal femur | 2016.09.20 | > 90 | 25.6 | 38.9 |
|  | Inguinal lymph node metastasis | 2016.10.16 |  | 93.4 | 62.5 |
|  | Pulmonary relapse 1 | 2017.07.10 |  | 47.8 | 64.7 |
|  | Pulmonary relapse 2 | 2018.11.12 |  | 112.4 | 57.6 |
| **P3** | Osteosarcoma left femur | 2017.07.24 | > 90 | 52.9 | 34.6 |
|  | Pulmonary relapse 1 | 2018.03.12 |  | 110.5 | 41.1 |
|  | Pulmonary relapse 2 | 2018.10.11 |  | 191.5 | 61.5 |
| **P4** | Osteosarcoma left femur | 2012.10.25 | 30 | 56.3 | 50.0 |
|  | Osseous relapse, left fibula | 2014.10.14 |  | 46.8 | 51.8 |
|  | Osseous relapse, rib | 2017.02.01 |  | 77.6 | 62.8 |

**Supplementary Table 3.** **Multivariate analysis between response neo-adjuvant chemotherapy and clinical and immunologic characteristics of OS samples at diagnosis.**

| **Parameter** | **Count** | **P Value** | **Logworth** | **FDR P Value** | **FDR Logworth** | **Effect Size** |
| --- | --- | --- | --- | --- | --- | --- |
| Gender | 21 | 0.83498 | 0.07832 | 0.997646 | 0.00102 | 0.04545 |
| Age at diagnosis | 21 | 0.99765 | 0.00102 | 0.997646 | 0.00102 | 0.00064 |
| Location | 21 | 0.12083 | 0.91783 | 0.402763 | 0.39495 | 0.50591 |
| Path Subtype | 21 | 0.19327 | 0.71384 | 0.431909 | 0.36461 | 0.52549 |
| Spread at diagnosis | 21 | 0.21595 | 0.66564 | 0.431909 | 0.36461 | 0.26594 |
| NLR at diagnosis | 21 | 0.06639 | 1.17788 | 0.331966 | 0.47891 | 0.4006 |
| TINs | 20 | 0.30377 | 0.51746 | 0.506276 | 0.29561 | 0.22996 |
| NETs (%) | 20 | *** 1.55E-07 | 6.80958 | 1.55E-06 | 5.80958 | 1.17315 |
| TILs | 19 | 0.92017 | 0.03613 | 0.997646 | 0.00102 | 0.02299 |
| No. of CD8 T cells | 19 | 0.83129 | 0.08025 | 0.997646 | 0.00102 | 0.04888 |

**Supplementary Table 4. Parametric survival fit, for overall survival of OS patients with local disease at diagnosis**

| **Event evaluated** | **Parameter** | **Count** | **FDR P Value** | **FDR Logworth** |
| --- | --- | --- | --- | --- |
| Overall survival | CD8s | 16 | 0.00000 | 89.222 |
|  | NETs | 16 | 0.00009 | 4.041 |
|  | TILs | 16 | 0.06312 | 1.2 |
|  | TINs | 16 | 0.14171 | 0.849 |
|  | NLR | 16 | 0.14171 | 0.849 |
| Progression-free survival | TILs | 16 | 0.00283 | 2.548 |
|  | CD8s | 16 | 0.04050 | 1.393 |
|  | TINs | 16 | 0.04227 | 1.374 |
|  | NETs | 16 | 0.09118 | 1.040 |
|  | NLR | 16 | 0.43205 | 0.364 |
